# Molecular and Biochemical Analysis of the Estrogenic and Proliferative Properties of Vitamin E Compounds

**DOI:** 10.3389/fonc.2015.00287

**Published:** 2016-01-05

**Authors:** Farid Khallouki, Philippe de Medina, Stéphanie Caze-Subra, Kerstin Bystricky, Patrick Balaguer, Marc Poirot, Sandrine Silvente-Poirot

**Affiliations:** ^1^INSERM UMR 1037, Cancer Research Center of Toulouse, University of Toulouse III, Toulouse, France; ^2^Université Paul Sabatier, Toulouse, France; ^3^Institut Claudius Regaud, Toulouse, France; ^4^Laboratoire de Biologie Moléculaire Eucaryote, CNRS, Toulouse, France; ^5^Université de Montpellier, Montpellier, France; ^6^INSERM U1194, Institut de Recherche en Cancérologie de Montpellier, Montpellier, France

**Keywords:** estrogen receptor alpha, estrogen receptor beta, vitamin E, molecular modeling, gene transcription, HMG-CoA reductase, breast cancer, proliferation

## Abstract

Tocols are vitamin E compounds that include tocopherols (TPs) and tocotrienols (TTs). These lipophilic compounds are phenolic antioxidants and are reportedly able to modulate estrogen receptor β (ERβ). We investigated the molecular determinants that control their estrogenicity and effects on the proliferation of breast cancer cells. Docking experiments highlighted the importance of the tocol phenolic groups for their interaction with the ERs. Binding experiments confirmed that they directly interact with both ERα and ERβ with their isoforms showing potencies in the following order: δ-tocols > γ-tocols > α-tocols. We also found that tocols activated the transcription of an estrogen-responsive reporter gene that had been stably transfected into cells expressing either ERα or ERβ. The role of the phenolic group in tocol–ER interaction was further established using δ-tocopherylquinone, the oxidized form of δ-TP, which had no ER affinity and did not induce ER-dependent transcriptional modulation. Tocol activity also required the AF1 transactivation domain of ER. We found that both δ-TP and δ-TT stimulated the expression of endogenous ER-dependent genes. However, whereas δ-TP induced the proliferation of ER-positive breast cancer cells but not ER-negative breast cancer cells, δ-TT inhibited the proliferation of both ER-positive and ER-negative breast cancer cells. These effects of δ-TT were found to act through the down regulation of HMG-CoA reductase (HMGR) activity, establishing that ERs are not involved in this effect. Altogether, these data show that the reduced form of δ-TP has estrogenic properties which are lost when it is oxidized, highlighting the importance of the redox status in its estrogenicity. Moreover, we have shown that δ-TT has antiproliferative effects on breast cancer cells independently of their ER status through the inhibition of HMGR. These data clearly show that TPs can be discriminated from TTs according to their structure.

## Introduction

**Figure 1:**
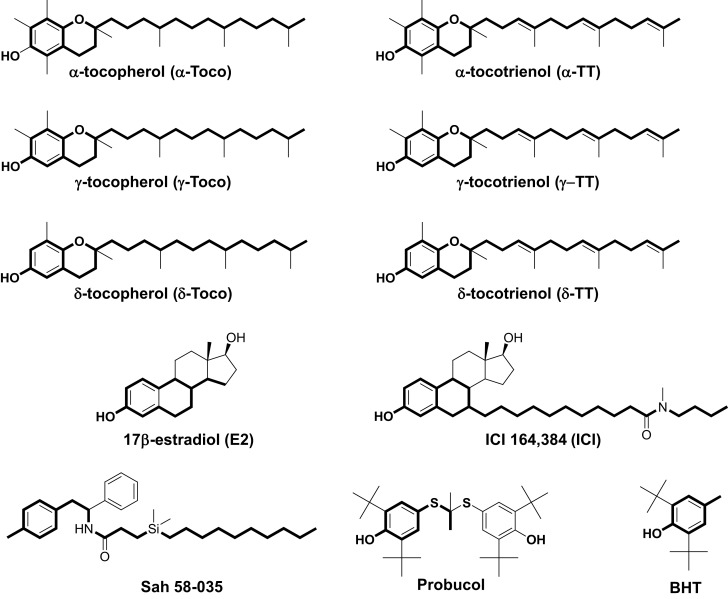
**Chemical structures of α-, γ-, and δ-tocopherol; α-, γ-, and δ-tocotrienol; 17β-estradiol; ICI 164,384; Sah 58-035; probucol; and butylated hydroxytoluene (BHT)**. Boldface type indicates the part of the molecules that are superimposable.

Vitamin E was first characterized in wheat germ oil and lettuce in 1922 (1). Vitamin E compounds are also known as tocols and include eight structurally related forms separated into two groups: tocopherols (TPs), in which the isoprenoid side chain is saturated, and tocotrienols (TTs), in which the side chain is unsaturated. The α-, β-, γ-, and δ-TP and -TT isomers are named according to the number of methyl groups on the chromanol ring at the 3, 5, and 7 positions (Figure 1). Vitamin E compounds have been extensively used in pharmacological studies due to their antioxidant properties; however, a major difference exists between TPs and TTs. TTs are potent down regulators of both HMG-CoA reductase (HMGR) and the isoprenoid-cholesterol biosynthesis pathway, and reduced cancer cell proliferation (2). In addition, TTs have been reported to induce cell cycle arrest and inhibit NFκB pathways and angiogenesis (3–6). Compared to TTs, much more is known about the effects of TPs. α-TP is quantitatively the major form of vitamin E found in humans and animals (7), whereas other TPs are present in various fat oils, such as palm oil (8) and argan oil (9). Many studies have focused their attention on vitamin E succinate (VES), a synthetic derivative of α-TP in which the hydroxyl phenol is esterified through succinylation. In contrast to α-TP, VES displays antiproliferative properties through an as-yet undefined mechanism *in vitro* and *in vivo* (10) but does not have antioxidant properties due to the esterification of the phenolic group. Vitamin E are fat-soluble antioxidants, and numerous studies have proposed that they can help in preventing or modulating diseases associated with oxidative stress, such as cardiovascular diseases (11, 12), neurodegenerative diseases (13), and cancers (14). Despite this, clinical trials have failed to establish any preventive effects of α-Toco on cardiovascular diseases and cancer (15–17). Recently, however, it was reported that dietary administration of δ- and γ-TP inhibited tumorigenesis in an animal model of estrogen receptor (ER)-positive but not human epidermal growth factor receptor (HER-2)-positive breast cancer (18). Parallel to this observation, a number of studies have shown that vitamin E, as an antioxidant, may interfere with the pharmacological action of some anticancer drugs, which rely on reactive oxygen species production as part of their mechanism of action (19). This is the case for the anticancer drug tamoxifen and other selective antiestrogen-binding site (AEBS) ligands such as tesmilifene, developed for the treatment of breast, lung, and prostate cancers (20–22), all of which have their antiproliferative and proapototic activities blocked by α-TP (23–27). α-TP has also been shown to inhibit the lipoperoxydation of cholesterol, blocking the production of the prodifferentiation and proapototic cholesterol-5,6-epoxides that have been identified as mediators of tamoxifen activity in breast cancer cells (21, 26, 28, 29). These data suggest that the intake of α-TP during prophylactic or curative treatment could impair the clinical outcome of patients treated with tamoxifen. In fact, many patients undergoing breast cancer treatment are known to take antioxidant dietary supplements, which may have a negative impact on their clinical outcome (30).

Tocopherol and TT contain structural determinants such as a phenol group, a cyclic structure, and long hydrophobic side chains that make them possible ligands for ERs (31). One study reported them to be weak modulators of ERβ but curiously found that they did not affect ERα activity (32). ERs are nuclear receptors (NRs) that mediate the biological effects of estrogens. They influence many physiological processes, including not only reproductive functions but also hormone-dependent cancers, cardiovascular health, bone integrity, immunity, cognition, and behavior (33, 34). The present study aimed to reevaluate the impact of TP and TT on ER-dependent transcriptional activity and breast cancer cell proliferation.

## Materials and Methods

### Chemicals

[^3^H]-17β-estradiol and [^14^C]-HMG-CoA were purchased from GE Healthcare (UK). ICI 182,780 was from Tocris (UK). TPs and TTs were from Merk-Millipore (USA) or were kindly provided by Dr. Abdul Gapor (Kuala Lumpur, Malaysia); other compounds and chemicals were from Sigma-Aldrich (USA). All solvents were from Prolabo (France).

### Synthesis of δ-Tocopherylquinone

A solution of gold III chloride (0.28 g; 0.92 mmol) dissolved in water (1 ml) was added dropwise to a solution of δ-Toco (0.36 g; 0.89 mmol) dissolved in ethanol (9 ml). The mixture was stirred in the absence of light for 2 h at room temperature. The solution was then evaporated and the solid residue was resuspended in dichloromethane and filtered. The organic layer was washed three times with water, dried over magnesium sulfate, filtered, and evaporated to dryness. The orange oil was purified by reverse phase HPLC (Ultrasep ES 100 RP 18, 250 × 8 mm, 6.0 μm, using acetonitrile for 10 min, linear gradient of 100% acetonitrile to 100% MeOH for 60 min; flow rate = 1 ml/min) and yielded a pure colorless oil product. MS: DCI (NH_3_), MH^+^ = 419; TLC Silica: Rf (CHCl_3_): 0.18; HPLC, Rt = 30 min; UV: λ_max_ = 260 nm.

### Molecular Structure Analysis

Computational chemical calculations were performed on a Silicon Graphics Indigo workstation using Insight II version 2000 (Accelrys, San Diego, CA, USA). Minimal energy conformations were calculated using the Discover module (2.9.7/95.0/3.0.0) with the CVFF force field. Van der Waals volumes and van der Waals volume intersections were determined using the Search-Compare module version 95.0 (Accelrys). We first compared the structure of α-tocopherol (δ-TP) with that of ICI 164,384. Superimposition was carried out between the energy minimized structure of α-TP and ICI 164,384 in the conformations adopted in the crystallographic structure of ERβ-ICI 164,384 (35) (Protein Data Bank 1HJ1). Superimposition was conducted using the diphenylethane part of α-TP that was superimposed carbon to carbon onto the steroidal backbone of ICI 164,384. The van der Waals volumes of α-TP and ICI 164,384 were also compared and the percentage of superimposition was calculated by measuring the ratio of the intersection of the van der Waals volume of ICI 164,384 with the van der Waals volume of α-TP.

### Estrogen Receptor-Binding Assay

Estrogen receptor-binding experiments with [^3^H]17β-estradiol were conducted exactly as reported in a previously published paper using extracts from Cos-7 cells transfected with expression vectors encoding human ERα and ERβ (36).

### Molecular Modeling with Estrogen Receptors

δ-tocotrienol (δ-TT), generated as described above, was prepositioned in the 4-hydroxytamoxifen (OHT)-ERα ligand-binding domain (LBD) crystal structure (Protein Data Bank 3ERT) (37) using the Search-Compare module of Insight II (Accelrys). The superimposition of OHT and δ-TT was carried out as described previously (38). Once prepositioned, OHT was unmerged from the OHT-ERα complex and deleted, and δ-TT was then merged to the receptor. The resulting complex was submitted to energy minimization using 250 steps of the steepest descent followed by a conjugated gradient until the root mean square gradient was <0.001 kcal/mol/Å. A distant-dependent dielectric term (ϵ = *r*) and a 20-Å non-bonded cutoff distance were chosen, whereas the hydrogen bond involved in the conformation of the α helices was preserved by applying a generic distance constraint between the backbone oxygen atoms of residue *i* and the backbone nitrogen atoms of residue *i* + 4, excluding prolines. This was performed using the Discover calculation engine with the CVFF force field (Insight II version 2000.1; Accelrys). The minimized coordinates of the receptor were then used as the starting point for 100 ps at 300 k using the Verlet algorithm whereas the constraint used during minimization was maintained. The resulting conformation was then further minimized using 250 steps of the steepest descent followed by a conjugated gradient until the root mean square gradient was <0.001 kcal/mol/Å.

### Reporter Cell Lines and Luciferase Assay

MELN cells were established by transfecting ER(+) MCF-7 cells with the ERE-β-globin-tk-Luc-SV-Neo plasmid (36). HELN cells were generated by transfection of ER(−) HeLa cells with this plasmid. The HELN-ERα, HELN-ERβ, HELN-ΔAB-ERα, and HELN-ΔAB-ERβ cell lines then underwent a second transfection with the corresponding pSG5-puro plasmids (pSG5-ERα-puro, pSG5-ERβ-puro, pSG5-ΔAB-ERα-puro, and pSG5-ΔAB-ERβ-puro, respectively) and expressed wild-type or mutated ERα or ERβ (39, 40). Mutated ERα or ERβ have been deleted for the AB domain which possesses a ligand-independent activation function (AF1). Comparison of the activities toward hERα and hERβ with the truncated ΔAB-ERα and ΔAB-ERβ provides a powerful model to identify partial ER agonists (requiring ligand-independent AF-1 to induce maximal ER activation). MELN and HELN cells expressed luciferase in an estrogen-dependent manner. Cells were grown routinely in DMEM growth medium supplemented with 5% FBS (Gibco BRL, Life Technologies, Cergy pontoise, France). Cells were incubated at 37°C in a humidified 5% CO_2_ incubator. For experiments, cells were grown for 5 days in phenol red-free medium, containing 6% dextran-coated charcoal-treated FCS (DCC-FCS) with penicillin–streptomycin. Medium was changed after 2 days. On day 5, cells were treated or not with the compounds, which were dissolved in ethanol. For each condition, 15 × 10^3^ cells were seeded per well in 12-well plates and treated, as described above, for 8 h in a final volume of 0.5 ml. At the end of the treatment, cells were washed with PBS and lysed in 150 μl lysis buffer (Promega, Charbonnières, France). Luciferase activity was measured using the luciferase assay reagent (Promega), according to the manufacturer’s instructions. Protein concentrations were measured using the Bradford technique (41) to normalize the luciferase activity data. For each condition, average luciferase activity was calculated from the data of three independent wells.

### Cell Extracts and Western Blots

MCF-7 cells were grown in 12-well plates and treated as indicated, then washed with PBS, and collected by centrifugation. Total cell lysates were prepared by resuspending the cells from each well in 100 μl lysis buffer (50 mM Tris pH 6.8, 2% SDS, 5% glycerol, 2 mM EDTA, 1.25% β-mercaptoethanol, 0.004% Bromophenol blue). Samples were boiled for 20 min at 95°C and cleared by centrifugation at 12,000 × *g* for 10 min. Protein concentration was determined by the Amido schwartz assay when samples contained SDS. Samples were subjected to PAGE on a 10% SDS-polyacrylamide gel in 25 mM Tris–HCl, 200 mM glycine, pH 8.3, 0.1% SDS, and proteins were then transferred onto a nitrocellulose membrane. Western blot analysis was performed as previously described (42) using rabbit polyclonal ERα antibodies diluted to 1 μg/ml (HC20 or H-184 Santa Cruz Biotechnology, Inc.) and the mouse antihuman glyceraldehyde 3-phosphate dehydrogenase (1:1,000). Visualization was achieved with an Enhanced Chemiluminescence Plus kit (Perkin Elmer) and luminescence was measured by either autoradiography or using a PhosphorImager (Storm 840; GE Healthcare).

### Cell Proliferation Assay

MCF-7 (ER(+)), T47D (ER(+)), and MDA-MB-231 (ER(−)) cell lines were from ATCC. Cell lines were maintained at 37°C in a humidified incubator in a 5% CO_2_-enriched atmosphere in T-75 flasks. MCF-7, T47D, and MDA-MB-231 cells were grown routinely in phenol red RPMI 1640 medium supplemented with 5% FBS (Gibco BRL, Life Technologies, Cergy Pontoise, France) and with penicillin–streptomycin. Cells were grown for 24 h before treatment in phenol red-free medium containing 5% DCC-FCS. Cells were seeded into 96-well plates at 2000 cells/well. Treatment media (150 μl/well) was added on the following day and replaced at 48-h intervals until the end of the experiment. Cell density was measured via the sulforhodamine B method (43) after 0, 2, 4, 6, and 8 days. The absorbance of SRB was measured directly at 490 nm in the 96-well plates using a multiskan^®^ multisoft reader from Labsystem.

### Determination of HMG-CoA Reductase Activity in Cell Extracts

The microsomal fraction of MCF-7, T47D, and MDA-MB-231 cells was prepared as previously described (44). HMGR activity was determined using the procedure first described by Brown et al. (45): 100 μg of microsomal protein was suspended in 0.1M potassium phosphate buffer pH 7.5 containing 20 mM glucose-6-phosphate, 2.5 mM NADP^+^, 1 unit of glucose-6-phosphate dehydrogenase, 5 mM dithiothreitol and 0.2 μCi [^14^C]-HMG-CoA. The reaction was stopped after 3 h by the addition of 25 μl 6 N HCl. Mevalonate was converted to lactone by standing at 37°C for 30 min, then extracted into 5 ml ethyl acetate, and brought to dryness by evaporative centrifugation. The sample was dissolved in 50 μl ethyl acetate and fractionated by silica thin-layer chromatography with toluene:acetone (1/1). Mevalonolactone was identified by comigration with authentic mevalonolactone visualized by iodine vapor staining and quantified storm analysis.

### Statistical Analysis

Values are the mean ± SEM of three independent experiments, each carried out in duplicate. Statistical analysis was made by two-way ANOVA, where appropriate (Prism 6, GraphPad Software, Inc., San Diego, CA, USA). ***P < 0.001; **P < 0.05; *P < 0.01; ns: not significant.

## Results

### TP and TT Share Structural Similarities with Estrogen Receptor Ligands

**Figure 2:**
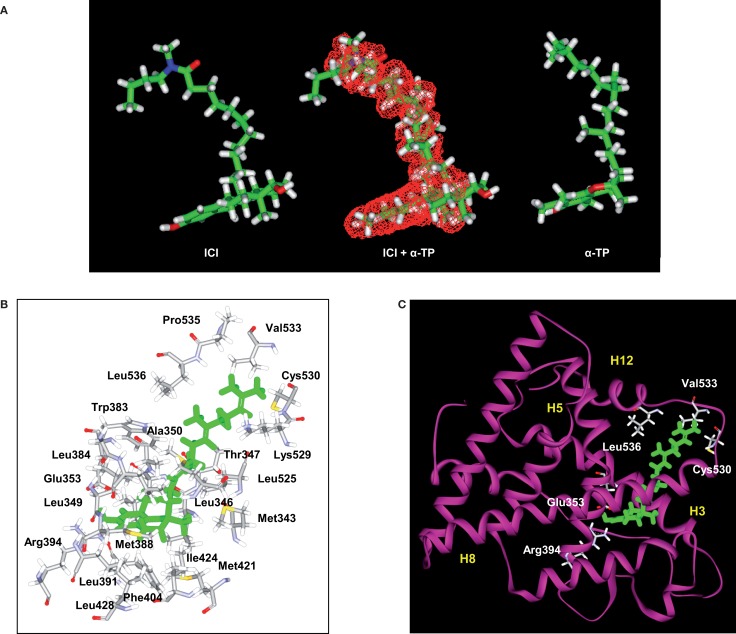
**Structural analyses of α-TP alone and δ-TP docked with ERα**. **(A)** Three-dimensional structures of the conformations of ICI 164,384 (ICI) (left), taken in the crystallographic structure of rat ERβ-ICI 164,384, and the calculated minimal energy conformation of α-tocopherol (α-TP) (right). The calculated minimal energy was carried out using the Discover module of Insight II (version 2000), as described in Section “Materials and Methods.” An overlay of α-TP and ICI (center), as well as van der Waals volume calculations and intersection measurements, was carried out using the Search-Compare module. The van der Waals volume intersection is depicted as the red grid and illustrates the structural similarities between α-TP and ICI. The van der Waals volumes of α-TP and ICI are 406.57 and 468.77 Å^3^, respectively. Sixty-three percent of the van der Waals volume of α-TP is in common with that of ICI. **(B)** Amino acids that interact with δ-TT: cross-sectional view of δ-TT within the ERα ligand-binding domain. Green: δ-TT; gray: carbon atoms; white: hydrogen atoms; red: oxygen atoms; blue: nitrogen atoms; and yellow: sulfur atoms. **(C)** Ribbon representation of the molecular model of δ-TT bound to ERα. δ-TT is drawn in stick form and colored in green. Helical elements of the ER are numbered (H3, H5, H8, and H12) and colored in yellow.

In previous studies, we used a pharmacophore approach to identify new targets for known drugs to explain some of their pharmacological properties (36, 38, 42, 46–48). We applied this approach to vitamin E compounds. The secondary structures of TPs, TTs, 17β-estradiol, ICI-164,384, Sah 58-035, probucol, and butylated hydroxytoluene (BHT) are shown in Figure 1. Tocols are phenolic compounds with a long hydrophobic side chain that are similar to ER ligands, such as ICI 164,384 or Sah 58-035, when drawn in a two-dimensional representation (36) (Figure 2A). This similarity was confirmed by comparison of the active structure of ICI 164,384 cocrystallized with ER-β with a minimal energy conformation of α-TP in a three-dimensional representation (Figure 2A). The van der Waals volumes of α-TP and ICI 164,384 were 406.57 Å and 469 Å, respectively (Figure 2A). Superimposition of the compounds is shown in Figure 2A and reveals that ICI 164,384 and α-TP share a common volume of 254.07 Å, which represents 63% of the van der Waals volume of α-TP. The hydrophobic side chain of both compounds gives a perfect superimposition, with the exception of the ultimate ethyl group of the side chain of ICI 164,384. This shows that the molecular volume defined by α-TP lies within the ligand-accessible volume of the ER and that the orientation of the hydrophobic side chain of tocols corresponds to that of the aliphatic site chain of ICI 164,384 and Sah 58-035. Altogether, these data are consistent with a direct interaction of α-TP with the ER.

### Tocols Are Ligands for ERα and ERβ

We next investigated whether α-, γ-, and δ-TPs and -TTs interact with the two human ER subtypes (ERα and ERβ) by conducting competition experiments with tritiated 17β-estradiol [^3^H]-E2 (Table 1). The tocols bound to ERα and ERβ with the following order of affinity (highest to lowest): δ-tocols > γ-tocols > α-tocols. Thus, increasing the hindrance of the phenol group by increasing the number of methyl groups led to a decrease in affinity for both ERs. The oxidized product of δ-TP, δ-tocopherylquinone (δ-TPQuin), did not bind to the ERs, highlighting the importance of the phenol group in ER interaction (Table 1). The phenolic antioxidants, BHT, and probucol, had no detectable affinity, probably because of the presence of two bulky tertiobutyl substituents adjacent to the hydroxyphenol group and because of the absence of a hydrophobic side chain. Interestingly, the IC_50_ values obtained for the TP corresponded to the concentrations they were tested on cell lines *in vitro* (100–500 μM) (23, 24, 26, 27, 49–53). These data show that tocols are ligands for both ERα and ERβ.

**Table 1:** **ER binding experiments**.

	ERα (IC_50_)	ERβ (IC_50_)
α-TP	453 ± 30 μM	431 ± 21 μM
γ-TP	227 ± 28 μM	215 ± 16 μM
δ-TP	118 ± 15 μM	98 ± 12 μM
α-TT	412 ± 22 μM	388 ± 32 μM
γ-TT	203 ± 25 μM	205 ± 18 μM
δ-TT	96 ± 6 μM	91 ± 7 μM
Probucol	N.M.	N.M.
BHT	N.M.	N.M.
δ-TPQuin	N.M.	N.M.

*Extracts from cos-7 cells transfected with expression vectors encoding human ERα and ERβ were incubated with 2 nM [^3^H]-E2 and different concentration of tocols ranging from 1 μM to 1 mM. IC_50_ values were determined using the iterative curve-fitting program GraphPad prism version 5 (GraphPad Software). N.M., not measurable*.

### Molecular Modeling of the δ-TP-ERα Complex

The ability of tocols to interact with ERs raised the question of the molecular consequences of this interaction. In the absence of a crystal structure of the tocol–ER complex, we investigated this issue through molecular modeling. Figures 2B,C show the chemical interactions between δ-TP and ERα. Interestingly, the phenol group of δ-TP inhabited the LBD in a similar fashion as E2: the hydroxyl group interacted with Glu-353 and Arg-394. The phenyl part of the chromanol group produced a T-shaped interaction with the phenyl side chain of Phe-404 and had van der Waals contacts with the methyl groups of Leu-391 and Leu-384. These data show that the chromanol backbone of δ-TP can occupy the same cavity as E2 or diethylstilbestrol (37, 54). The side chain of δ-TP protruded into the 11β cavity of the LBD of ERα and produced multiple van der Waals interactions with hydrophobic amino acids, such as Ala-350, Leu-525, and Trp-383. The upper part of the side chain interacted with Val-533, Leu-536, Leu-539, Leu-540, and Met-543. These latter amino acids belong to helix H12, thus showing an interaction between the upper part of the side chain of δ-TP and helix H12 in this model, as was observed for Sah 58-035 (36). δ-TP established a van der Waals interaction with Met-421 but no interactions were detected with Leu-384, suggesting that they might not discriminate between the two ER subtypes, which are consistent with binding experiments. The docking of the more hindered α-tocols showed a loss of the interaction of the hydroxy phenolic group with Glu-353 and Arg-394, explaining their weaker affinity compared to δ-tocols. These data illustrate that tocols are accommodated well within the ER binding site in a similar way as that previously established with Sah 58-035 and auraptene (36, 42). This suggests that tocols can act as modulators of ERs rather than pure agonists.

### Tocols Are Partial Agonists for ER-Mediated Transcription

**Figure 3:**
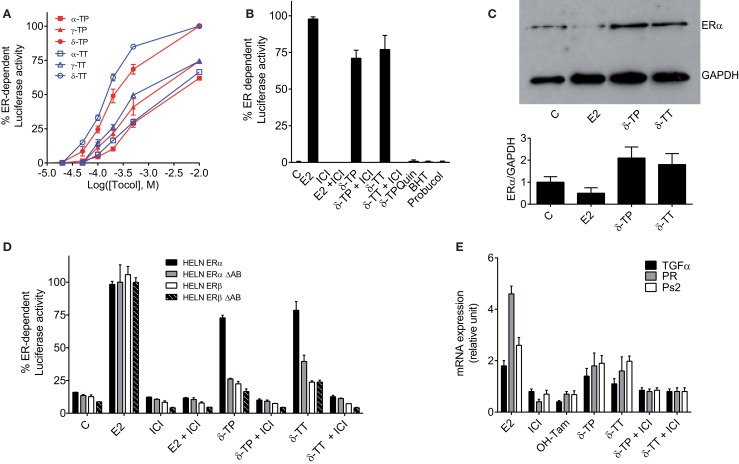
**Measurement of estrogenicity of tocols *in vitro***. **(A)** Dose–response curve of the effect of tocols on MCF-7 cells stably transfected with the ERE-β-globin-tk-Luc plasmid (MELN cells). Results are represented as the percentage of ER-dependent α-, γ-, and δ-TP and α-, γ-, and δ-TT luciferase activity obtained with 10 nM E2 and increasing concentrations of tocols ranging from 10 to 500 μM. **(B)** ER-dependent transcriptional modulatory activity of 500 μM δ-TP, δ-TT, δ-Tocopherolquinone (δ-TPQuin), butylated hydroxytoluene (BHT), and probucol in MELN cells. Cells were incubated with either 10 nM 17β estradiol (E2), 500 μM δ-TP or 500 μM δ-TT alone or in combination with 1 μM pure antiestrogen ICI 164,384 (ICI) or were incubated with 500 μM δ-TPQuin, 500 μM BHT or 500 μM probucol and assayed for luciferase activity. Data shown are the mean values ± SEM from three independent experiments. **(C)** δ-TP or δ-TT stabilized ER-α in MCF-7 cells. MCF-7 cells were cultured as described in Section “Materials and Methods” and treated with either solvent vehicle (EtOH), 100 nM E2, 500 μM δ-TP or 500 μM δ-TT for 3 h. MCF-7 extracts were analyzed for the presence of ERα by western blotting using GAPDH as a control. Visualization was achieved with an Enhanced Chemiluminescence Plus kit and fluorescence was measured by either autoradiography or using a PhosphorImager. The western blot shown is representative of three independent experiments. The ratio of ERα to GAPDH levels in each experiment was determined densitometrically and normalized to control value (taken to be 1). **(D)** Effect of δ-tocols on estrogen response element-dependent luciferase activity in HELN cells, which are HELA cells transfected with either fully functional ERα or ERβ or their mutated versions containing a deletion the AB domain (ΔAB). Cells were incubated with 10 nM 17β estradiol (E2), 500 μM δ-TP or 500 μM δ-TT alone or in combination with 1 μM pure antiestrogen ICI 164,384 (ICI) and assayed for luciferase activity. Data shown are the mean values ± SEM from three independent experiments. **(E)** δ-Tocols modulate the induction of endogenous genes under the control of ER. Cells were treated with solvent vehicle, 10 nM E2, 1 μM ICI, 1 μM OH-Tam. Cells were treated with 500 μM δ-tocols in the presence or in the absence of ICI. The relative expression of TGFα, PR, and Ps2 (TFF1) after 16 h was analyzed by quantitative RT-PCR. Data shown are the mean values ± SEM from three independent experiments.

The next set of experiments were designed to investigate whether tocols can modulate ER-dependent transcription, using MCF-7 cells stably transfected with a plasmid encoding an estrogen-responsive promoter fused to the luciferase gene (MELN cells) (36). Figure 3A shows that all the tocols tested stimulated luciferase transcription, with the best response obtained using 500 μM δ-TP and δ-TT, which resulted in 76.5 and 86.6% of the maximal ER-dependent response (taken as that obtained from treatment with 10 nM E2), respectively. Tocol-induced ER-dependent transcriptional activity was blocked in the presence of the ER antagonist ICI 164,384 (Figure 3B). As expected, compounds that were previously determined as non-ER ligands, such as δ-tocopherylquinone (δ-TPQuin), BHT, and probucol, did not stimulate the expression of luciferase (Figure 3B). We next established that δ-TP- and δ-TT-bound ERα was not degraded as was observed for ERα bound to E2 (Figure 3C). Thus, the effect of δ-TP and δ-TT on ER protein stability is similar to that of selective ER modulators, suggesting that tocols are not pure estrogens. δ-TP and δ-TT were also shown to activate ER-dependent luciferase activity through both ERα and ERβ, using HELN cells (HeLa cells transfected with the same plasmid as MELN cells, which encodes an estrogen-responsive promoter fused to the luciferase gene) (Figure 3D). In order to further characterize the agonistic properties of the δ-tocols, the HELN-ERα and -ERβ cell lines were used alongside the HELN-ΔAB-ERα and HELN-ΔAB-ERβ cell lines in which the N-terminal AF1 domain of the ERs (responsible for the majority of ER transactivation activity) is deleted (40). We observed that ERα-mediated transcriptional activation induced by the δ-tocols was strongly altered in the absence of the AF1 domain, whereas the loss of this domain did not significantly affect ERβ-mediated transcriptional activation induced by the δ-tocols (Figure 3D). To determine whether δ-TP and δ-TT can modulate the expression of endogenous E2-regulated genes as well as reporter genes, the expression of the progesterone receptor gene (PR), trefoil factor-1 (TFF1, Ps2), and transforming growth factor alpha (TGFα) was measured by quantitative RT-PCR in MCF-7 cells. Treatment of MCF-7 cells with δ-TP stimulated the transcription of TGFα (1.4-fold increase), PR (1.8-fold), and Ps2 (1.9-fold) (Figure 3E). The treatment of MCF-7 cells with δ-TT stimulated the transcription of TGFα (1.1-fold), PR (1.6-fold), and Ps2 (twofold). These results confirm that δ-TP and δ-TT can activate the transcription of endogenous genes that are known to be under the control of ERα.

### The Effect of δ-Tocols on the Proliferation of ER(+) and ER(−) Breast Cancer Cells

**Figure 4:**
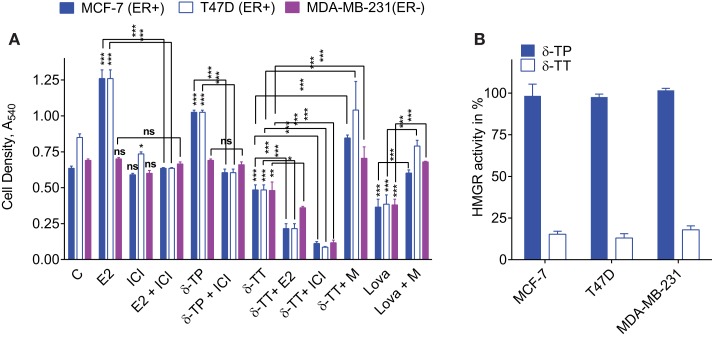
**Effect of tocol on ER(+) and ER(−) BC cell proliferation and HMG-CoA reductase activity**. **(A)** Effect of δ-tocols on estrogen and non-estrogen-regulated growth of MCF-7, T47D, and MDA-MB-231 cells. Cells were treated with solvent vehicle (C), 1 nM 17β estradiol (E2), or 500 μM δ-TP in the presence or absence of 1 μM ICI. Alternatively, cells were treated with 500 μM δ-TT in the presence or absence of 100 nM E2, 1 μM ICI, or 5 mM mevalonolactone (M). Cells were also treated with 30 μM lovastatin (Lova) in the presence or absence of 5 mM M, as described in Section “Materials and Methods.” Values are shown as means and vertical bars represent SEM. The data were analyzed by two-way ANOVA, followed by Bonferroni *post hoc* test. ***P < 0.001, **P < 0.01, and *P < 0.05 in comparison with control or bare-linked specific control. Ns: not significant. **(B)** Effect of δ-TT on hydroxymethylglutaryl coenzyme A reductase (HMGR) activity in MCF-7, T47D, and MDA-MB-231 cells. Cells were treated with 500 μM δ-TT or δ-TP, as described in Section “Materials and Methods.” Results are reported as the percentage of HMGR activity compared to solvent vehicle-treated cells. Data shown are the mean values ± SEM from three independent experiments performed in triplicate.

To investigate the effects of δ-TP and δ-TT on cell growth, ER(+) human BC cell lines (MCF-7 and T47D) and an ER(−) BC cell line (MDA-MB-231) were used. As shown in Figure 4A, δ-TP (500 μM) induced a significant stimulation of MCF-7 and T47D cell proliferation over a 6-day period, albeit to a lesser extent than E2 (10 nM), and had no impact on ER(−) MDA-MB-231 cells. Both δ-TP- and E2-induced stimulation of proliferation was blocked by the ER antagonist ICI 164,384, consistent with an ER-mediated event (Figure 4A). In contrast, δ-TT inhibited the proliferation of cells, and these effects were amplified in the presence of E2 or ICI 164,384. Only cotreatment of cells with mevalonolactone (M) protected all three cell lines from the inhibitory effects of δ-TT (Figure 4A). Mevalonolactone is known to reverse the mevalonate-isoprenoide pathway when HMGR is inhibited suggesting that δ-TT inhibited HMGR in BC cells as observed in other cell lines (2, 55–58). We found a similar effect using when cells where treated with lovastatin, a prototypical inhibitor of HMGR, and as expected, the inhibition of cell proliferation was reversed by mevalonolactone (Figure 4A). These differential actions of δ-TP and δ-TT are consistent with an inhibition of HMGR activity that was observed downstream of δ-TT but not δ-TP in these cell lines (Figure 4B). Altogether, these data show that δ-TP stimulates cell proliferation in a similar way to that of ER agonists while δ-TT inhibits cell growth, consistent with its capacity to down regulate HMGR.

## Discussion

In this paper, we report the identification of a new molecular target of vitamin E compounds that sheds light on their pharmacological potency and the potential risks related to their specific substructures. Using a ligand-structure based approach, we found that TPs and TTs are ER ligands and behave like partial agonists in ER-mediated transcriptional regulation of synthetic and endogenous genes. Therefore, they are phytoestrogens. Consistent with this data, vitamin E has been previously reported to increase the expression of estrogenic markers in breast biopsies of patients (53). We found that both the effects of the tocol derivatives on transcription and their affinity for ERα decreased with the number of methyl groups present on the phenol ring of the compounds, the most potent phytoestrogens being δ-TP and δ-TT. These data emphasize the importance of the accessibility of the OH phenolic group in establishing a productive interaction with the Glu353 and Arg394 residues in ERα. Molecular modeling studies suggested that the aliphatic side chain of tocols can occupy the 11β-cavity of the LBD, as observed for the side chains of steroidal and non-steroidal ER ligands (59, 60). The tocol side chain enables their interaction with helix H12 on the NR box-binding site (Figure 2B), consistent with an agonistic activity. The use of AF1 deletion mutants also demonstrated the requirement of the AF1 transactivation domain for δ-tocol activity and revealed that they act differently than E2 on ERα since they do not induce receptor degradation upon binding. Other phenolic antioxidants, such as BHT or probucol, did not display any estrogenic effects, which supports the observation that the estrogenic action of δ-TP was peculiar in its ER-binding activity. The presence of a bulky tertiobutyl group in the ortho position from the hydroxyl of the phenols in these compounds may explain this effect. Furthermore, the oxidated form of δ-TP had no estrogenic activity as a consequence of its loss of affinity for binding to the ER. This established that ER binding and ER-dependent transcriptional stimulation of tocols are dependent upon their reduced form status.

It is noteworthy that δ-tocols were found to stimulate TGFα expression *in vitro* in human breast cancer cells. TGFα can activate mitogenic pathways; so, this finding highlights the potential risk that these compounds could promote tumor growth. However, dietary administration of δ-TP was shown to protect against *N*-methyl-*N*-nitrosourea hormone-dependent tumorigenesis in Sprague-Dawley rats (18); therefore, based on the present data, it is now important to determine whether this effect is observable in different rodent species because potential selective ER modulator activity has been shown to induce responses in animal models that are not seen in humans (61).

In this paper, we report that TPs and TTs are agonists for both ER subtypes. We show that δ-TP stimulated the proliferation of ER-expressing cells, whereas TTs were potent inhibitors of cell proliferation irrespective of the cell’s ER status. This difference in activity could have resulted from the capacity of TTs to downregulate HMGR activity since it was reversed through addition of mevalonolactone, demonstrating the importance of the inhibition of the isoprenoide-cholesterol pathway in this effect (Figure 4A). Based on these data, we established that it is possible to distinguish between the action of TPs and TTs since although both TPs and TTs displayed antioxidant and ER stimulatory activity, and only TTs displayed an antiproliferative activity.

Altogether, these data have established that tocols are phytoestrogens and that their transcriptional modulation of ER must be taken into account to better understand their properties.

## Author Contributions

FK performed chemical, biochemical studies and analyzed data. PM performed biochemical studies and analyzed data; SC-S and KB performed biochemical and cellular experiments and analyzed the data; PB performed cellular experiments and analyzed the data; MP and SS-P designed the experiments, performed molecular modeling studies, analyzed the data, and wrote the paper.

## Conflict of Interest Statement

The authors declare that the research was conducted in the absence of any commercial or financial relationships that could be construed as a potential conflict of interest.
